# Editorial: Microplastics and microorganisms in the environment, volume II

**DOI:** 10.3389/fmicb.2024.1464294

**Published:** 2024-08-06

**Authors:** Xianhua Liu, J. Paul Chen, Lei Wang, Zongze Shao, Xiang Xiao, Jiao Wang

**Affiliations:** ^1^School of Environmental Science and Engineering, Tianjin University, Tianjin, China; ^2^Department of Civil and Environmental Engineering, National University of Singapore, Singapore, Singapore; ^3^College of Environmental Science and Engineering, Nankai University, Tianjin, China; ^4^Third Research Institute of the Ministry of Natural Resources, Xiamen, China; ^5^School of Oceanography, Shanghai Jiao Tong University, Shanghai, China; ^6^School of Energy and Environmental Engineering, Hebei University of Technology, Tianjin, China

**Keywords:** microplastics, microorganisms, interaction, biodegradation, virus

The pervasive use of plastics has led to the widespread presence of microplastics across our planet (Thompson et al., 2004; Wang et al., 2019). These tiny particles have been detected in Antarctic sea ice, the guts of marine animals inhabiting the deepest ocean trenches, and in drinking water worldwide. Microorganisms form the foundation of all life on Earth, and play essential roles in sustaining life through their various activities (Liu et al., 2021). Studying interactions between microplastics and microorganisms is of importance for a good number of reasons, such as encompassing environmental, ecological, human health, and socio-economic dimensions (Wang et al., 2021). For instance, identifying microorganisms capable of degrading microplastics can lead to formulation of sound remediation strategies, offering potential solutions to mitigate plastic pollution. Although significant progress has been made in the past few decades in understanding the relationship between microplastics and microorganisms in diverse environments, our comprehension of these interactions remains limited due to their inherent complexity.

The five papers in this virtual special issue (VSI) primarily focus on two topics: microbial degradation of microplastics and interactions between microplastics and viruses. The first topic involves identifying bacteria and microorganisms capable of efficiently degrading microplastics. In the environment, microplastics readily form microbe-rich plastispheres, implying that microbially mediated plastic degradation could be a viable solution to plastic pollution. A common approach to studying this involves using enrichment cultures to observe dynamic changes in microbial communities and identify microorganisms capable of degrading microplastics.

Olabemiwo et al. employed an innovative approach by using an improved Winogradsky Column (WC) to enrich plastic-degrading bacteria and genes in landfill soil from Connecticut. Their exciting results revealed new bacterial lineages with potential polyethylene (PE) degradation capabilities. Zhu et al. fed polystyrene foam to *T. molitor* larvae and investigated how this dietary change affected the gut bacterial community composition. This groundbreaking study addresses the research gap on anaerobic bacteria in *T. molitor* larvae's gut, offering a potential method to expand plastic biodegradation pathways and ultimately mitigate plastic waste accumulation. Pawano et al. utilized 16S metagenomic sequencing to assess the diversity of microbial communities enriched with polypropylene (PP), aiming to study the microbial communities involved in PP biodegradation. Zhang et al. collected plastic foam debris from the intertidal zone between April and October 2022 and analyzed the plastisphere community composition to evaluate the role of microorganisms in plastic waste biodegradation, which may influence the environmental fate of plastics either *in situ* or as they drift with water currents.

Current microbial studies on microplastics primarily focus on bacteria, fungi, and algae that colonize plastic surfaces, forming biofilms known as the “plastisphere” (Peng et al., 2022; Li et al., 2023). However, recent research has revealed that plastispheres also host a wide range of viruses, sparking interest in virology. Wang et al. provided a brief review of research on microplastics and viruses, covering the adsorption of viruses on both biotic and abiotic surfaces of microplastics and the factors influencing these interactions. They focused on the mechanisms by which microplastics affect viral toxicity, indicating that microplastics influence the transport, survival, and virulence of viruses. Given that viruses constitute a significant portion of the microbial world, the interaction between microplastics and viruses is a promising area of research with the potential to become a new focal point in the future.

In conclusion, these papers significantly advance our understanding of the interactions between microplastics and microorganisms, which is crucial for managing and predicting the risks associated with plastic pollution. However, several important topics remain underexplored, such as advanced analytical techniques, human health risk assessment, and bioremediation applications. Further research in all these areas can deepen our comprehension of the complex interactions between microplastics and microorganisms, ultimately contributing to the development of effective strategies for mitigating the environmental and health impacts of plastic pollution.

## Author contributions

XL: Conceptualization, Investigation, Resources, Writing – original draft, Writing – review & editing. JC: Writing – original draft, Writing – review & editing. LW: Writing – original draft, Writing – review & editing. ZS: Writing – original draft, Writing – review & editing. XX: Writing – original draft, Writing – review & editing. JW: Writing – original draft, Writing – review & editing.
